# Determinants of Chimeric Antigen Receptor (CAR) T Cell Success: In Vitro and In Vivo Preclinical Assessment

**DOI:** 10.3390/cancers18091412

**Published:** 2026-04-29

**Authors:** Michael K. Sheng, William J. Murphy

**Affiliations:** Department of Dermatology, University of California Davis School of Medicine, Sacramento, CA 95817, USA

**Keywords:** CAR T cells, immunotherapy, preclinical modeling, in vitro, in vivo, translational research

## Abstract

Background/Objectives: Chimeric antigen receptor (CAR) T cell therapy has profoundly transformed the cancer treatment landscape, achieving unprecedented clinical success in patients with hematological malignancies. However, challenges such as cytokine release syndrome, neurotoxicity, antigen escape, and limited efficacy in solid tumors remain, underscoring the need for robust preclinical modeling to evaluate novel CAR T cell products. Methods: This review provides a comprehensive overview of in vitro and in vivo preclinical modeling for CAR T cell functionality and toxicity assessment. We examine traditional experimental approaches and their limitations, discuss emerging technologies, and highlight how these strategies can be integrated to advance future CAR T cell therapies. Results: In vitro assays provide insights into efficacy but fail to model trafficking, dynamic immune cell interactions, and complex tumor microenvironments. In vivo mouse models allow for more complex physiological evaluation but are limited by species differences. Next generation platforms, such as patient-derived tumor organoids and organ- or multi-organ-on-a-chip microfluidics are emerging as potential tools to model CAR T cell therapy in physiologically relevant contexts and computational approaches are being increasingly used to develop novel CAR designs and predict patient responses. Conclusions: By integrating traditional experimental approaches with innovative technologies, the CAR T cell field is poised to generate more clinically relevant and predictive data thereby accelerating the development of safer, more effective, and personalized CAR T cell therapies.

## Simple Summary

Chimeric antigen receptor T cell therapy is an innovative new treatment that engineers a patient’s own immune cells to destroy cancer. While it has achieved incredible success in certain blood cancers and saved numerous lives, many patients do not respond, some experience serious side effects, and positive results in solid tumors have been limited. This review examines how researchers evaluate CAR T cell products before they reach patients, including traditional cell-based studies and animal models, as well as newer high-tech approaches. Understanding and improving these strategies may help researchers better predict safety and effectiveness and accelerate the development of more reliable and accessible CAR T cell therapies in the future.

## 1. Introduction

In the past decade, chimeric antigen receptor (CAR) T cell therapy has dramatically enhanced the cancer immunotherapy landscape, providing not only a highly efficacious alternative to conventional cancer treatments but also creating a biological blueprint that can potentially be harnessed to treat previously uncurable and hard-to-treat diseases.

In CAR T cell therapy, T cells are taken from a patient and genetically modified to express an artificial receptor, known as a “CAR,” enabling them to recognize tumor-associated antigens (TAAs) on tumor cells and mount a specific and powerful response against them. These CAR T cells are expanded for several weeks ex vivo before being reinjected back into the patient where upon TAA recognition they become activated, proliferate, and eradicate tumor cells. Unlike therapeutic antibodies and small molecules that dissipate and clear from circulation, CAR T cells persist quiescently even after tumor clearance, acting as a “living drug” that continuously monitors the body and reactivates should the tumor recur [1].

Clinical trials, such as ELIANA/ENSIGN, ELARA and ZUMA-1, have demonstrated the potent efficacy of CAR T cells in achieving remission and significantly improving overall survival (OS) in relapsed or refractory (R/R) B cell acute lymphoblastic leukemia (B-ALL) and R/R large B cell lymphoma (LBCL) [2,3,4]. In a direct comparative study, CAR T cell therapy outperformed chemotherapy in terms of remission and OS in patients with R/R B-ALL [5]. Furthermore, several matching-adjusted indirect comparison studies have associated CAR T cell therapies with improved OS, reduced risk of death, and fewer hospitalizations when compared to historical standard-of-care [6,7]. These findings support CAR T cell therapy not only as a viable treatment option but in certain contexts as a superior alternative to conventional treatments.

Despite clinical successes, CAR T cell therapy still faces significant hurdles, including variable patient responses, cytokine release syndrome (CRS), neurotoxicity, tumor heterogeneity, antigen escape, and limited efficacy in solid tumors [8]. Addressing these issues requires comprehensive preclinical modeling that involves robust in vitro and in vivo experimental design to better predict patient outcomes. This review examines these key aspects of CAR T cell preclinical modeling, highlighting current strategies, limitations, and emerging innovations aimed at improving translational predictability and advancing the next generation of CAR T cell therapies.

## 2. In Vitro Characterization of CAR T Cell Efficacy

After generation, CAR T cells are typically first evaluated in 2D in vitro assays to assess their phenotype, functionality, and persistence. Standard assays involve co-incubating CAR T cells with either bead- or plate-bound antigen(s) or immortalized antigen-expressing cell lines for 24 to 72 h [9]. This setup can be performed in single or repeated rounds of restimulation with antigenic targets. Repeated rounds of co-culture allow evaluation of the durability of the CAR T cell response and the effects of prolonged activation on phenotype, such as the development of T cell exhaustion [10]. Furthermore, repeated stimulations may better model clinical scenarios where tumor cells are not immediately eliminated but require prolonged immune control and persistence, which may also provide valuable insights into potential mechanisms of tumor resistance [11].

### 2.1. Key Experimental Parameters

During in vitro experimental co-culture, several variables must be considered from both the CAR T cells and the target antigen-expressing cells or plate-bound antigen. For the CAR T cell population, a key parameter is transduction efficiency. Assay reproducibility relies on consistent transduction efficiency. This can be controlled in part by determining whether the CAR T cells added consist of the total T cell population (including both transduced and non-transduced cells) or a purified population of transduced CAR-expressing T cells. Another important consideration is the number of donors used for CAR T cell generation and functional characterization. This is critical to ensure that the observed effects are robust and represent the broader patient population. CAR T cells derived from different donors can exhibit variability in phenotypic profiles, expansion and persistence, and functional efficacy (including cytotoxicity) even when assay conditions are the same. Therefore, evaluating CAR T cells from multiple donors improves the reliability and reproducibility of results and avoids overstating or understating functional outcomes based on a single donor [12,13]. Experiments involving multiple donors represent biological replicates while repeated experiments using CAR T cells generated from the same donor represent technical replicates. Additionally, manufacturing conditions, such as the cytokines used during ex vivo culture, the CD4:CD8 ratio, the media composition, and the culture duration, can influence CAR T cell phenotype and functional efficacy [14]. Maintaining consistent parameters and manufacturing conditions is essential for reproducibility of results.

For antigen-expressing target cell lines, key variables include the cell type or source, antigen stability (e.g., whether the antigen internalizes upon engagement), and intrinsic resistance such as expression of inhibitory receptor ligands. Antigen expression and density are also key parameters when using antigen-expressing cell lines or plate-bound antigen, respectively. During co-culture experiments, it is also important to test a range of effector-to-target (E:T) ratios, the ratio of CAR T cells to antigen-expressing target cells, or to test the dose response of plate-bound antigen to assess the sensitivity of CAR signaling and downstream effector function.

Additional experimental variables during the setup include the co-culture plate format. While direct contact assays performed in standard 96-well plates allow CAR T cells to interact directly with antigen-expressing cells and reveal maximal cytotoxic and effector ability, Transwell plates physically separate effector and target cells, therefore enabling discrimination between cell contact-dependent cytotoxicity and tumor cell death mediated by soluble factors such as cytokines. Transwell assays may also provide insight into CAR T cell migration and trafficking behaviors in vitro [15]. Last, but importantly, media composition is another key factor, especially whether exogenous cytokines are included during the co-culture. CAR T cell activation leads to the release of pro-inflammatory cytokines and the further addition of serum or exogenous cytokines can further influence T cell expansion, differentiation, and effector function.

**Figure 1:**
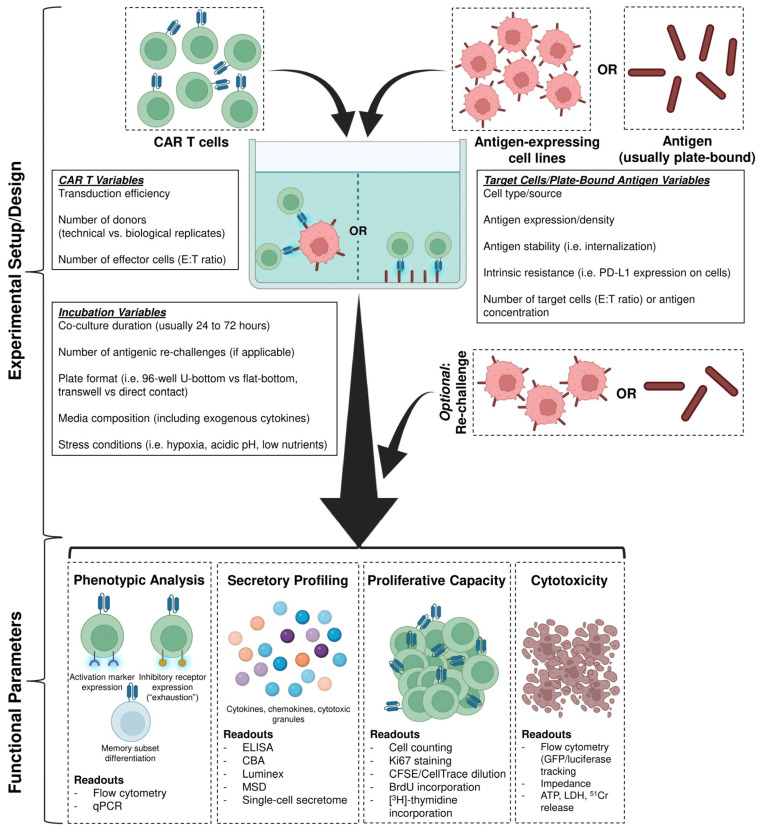
In vitro modeling of CAR T cells. For initial assessment CAR T cells are evaluated in 2D in vitro assays by co-incubating with either immortalized antigen-expressing cell lines or either bead- or plate-bound antigen(s) for around 24 to 72 h. This setup can be performed in a single round or repeated rounds of co-culture with antigenic targets. Listed are key variables on CAR T cells, target cells/plate-bound antigen, and incubation conditions, influencing assay design and measurements. After co-culture CAR T cell characterization typically focuses on four key parameters: (1) phenotypic analysis (activation, exhaustion, and memory differentiation), (2) secretory profiling (cytokines, chemokines, and cytotoxic granules), (3) proliferative capacity, and (4) cytotoxicity.

After co-culture, CAR T cell in vitro characterization focuses on four key parameters: (1) phenotypic analysis (activation, exhaustion, and memory markers), (2) secretory profiling (cytokines, cytotoxic granules, and chemokines), (3) proliferative capacity, and (4) cytotoxic function (Figure 1).

### 2.2. Phenotypic Analysis

CAR engagement with antigen results in robust T cell activation, which can be evaluated by upregulated surface expression of activation markers such as CD25, CD69, CD71, and HLA-DR [16]. Conversely, increased expression of inhibitory receptors such as PD-1, LAG-3, TIGIT, TIM-3, and 2B4 may indicate emerging or greater T cell exhaustion, which is associated with poorer clinical outcome [17,18,19,20]. These markers can be measured either by flow cytometry or qPCR. Additionally, flow cytometry enables assessment of memory subset differentiation using delineation markers such as CD62L, CCR7, CD45RA, and CD45RO to classify T cells into their respective subsets: naïve (T_N_, CD62L+CCR7+ CD45RA+CD45RO-), stem cell memory (T_SCM_, CD62L+CCR7+CD45RA+CD45RO+), central memory (T_CM_, CD62L+CCR7+CD45RA-CD45RO+), effector memory (T_EM_, CD62L-CCR7-CD45RA-CD45RO+), and effector (T_EFF_, CD62L-CCR7-CD45RA+CD45RO-), allowing for monitoring of the phenotypic shift towards progressive terminal differentiation following antigen exposure [21]. This is especially important as studies have shown that CAR T cells enriched in T_SCM_ and T_CM_ compartments are linked to enhanced persistence and long-lasting anti-tumor efficacy [22,23].

### 2.3. Secretory Profiling

Supernatants collected from co-cultures can be analyzed for cytokines, cytotoxic granules, and chemokines, using enzyme-linked immunosorbent assay (ELISA), cytokine bead arrays (CBAs), or high-throughput multiplex platforms such as Luminex, Meso Scale Discovery (MSD), or single-cell secretome technologies [24,25]. The secretion of key inflammatory cytokines, including IL-2, IFN-γ, and TNF, and cytotoxic granules, including granzyme B and perforin, is indicative of functional CAR T cell activation and cytolytic potential [24,26]. However, while elevated cytokine secretion may indicate potent effector function, it may also correlate clinically with an increased risk of CRS, ICANS, or tumor lysis syndrome (TLS) [27]. In addition, chemokines such as CCL19, MIP-1α, and SDF-1α may indicate the potential of CAR T cells to recruit other immune populations, especially innate immune cells [28].

### 2.4. Proliferative Capacity

The ability of CAR T cells to expand upon antigen encounter is a critical indicator of therapeutic efficacy. Clinically, greater in vivo CAR T cell expansion has been associated with complete responders, while limited expansion is more often observed in non-responders or partial responders [29]. In vitro, proliferative capacity can be assessed directly by counting total cell numbers poststimulation or by [3H]-thymidine incorporation assays. Alternatively, flow cytometry approaches can also be used, including detection of proliferation markers, such as Ki-67, or BrdU incorporation, as well as tracking dye dilution methods using CFSE or CellTrace Violet [30].

### 2.5. Cytotoxic Function

Cytotoxicity can be measured both directly and indirectly. Direct methods include quantifying target cell death by flow cytometry using viability dyes (e.g., propidium iodide, 7-AAD) or apoptosis markers (e.g., annexin V) to distinguish between live, apoptotic, and dead target cells, or by engineering target cells to express fluorescent proteins (e.g., GFP) or luciferase, then monitoring the loss of these signals over time as an indicator of cell death [31]. Indirect methods include impedance-based killing assays (e.g., xCELLigence) or measuring the release of intracellular contents such as ATP, lactate dehydrogenase (LDH), or chromium-51 from lysed cells [31]. These assays provide multiple functional readouts to evaluate the ability of CAR T cells to kill antigen-expressing tumor target cells.

### 2.6. Limitations of Preclinical In Vitro Assessment

While in vitro assays serve as a useful first pass in evaluating CAR T cell effector function and proliferative capacity upon antigen encounter, they fall short in replicating complex in vivo physiological conditions (Table 1). Standard co-culture assays create an artificial environment where CAR T cells are placed in close immediate proximity with tumor cells overexpressing the targeted antigen. In reality, CAR T cells must circulate systemically, traffic to the tumor site, and infiltrate the TME, where its functionality is influenced by physical, immunological and metabolic barriers.

In solid tumors, antigen expression may not be restricted to malignant cells and are often expressed at varying levels on normal tissues, increasing the risk of on-target, off-tumor (OTOT) toxicity, a phenomenon observed in multiple clinical trials [32]. Additionally, while tumor cell lines used in co-culture assays typically express uniformly high levels of antigen, patient tumors are generally far more heterogeneous and may exhibit low antigen density, which can reduce CAR T cell activation and cytotoxicity [33].

A major limitation of in vitro modeling is the absence of an immunosuppressive TME. In vivo, CAR T cells must contend with the presence of regulatory T (Treg) cells, myeloid-derived suppressor cells (MDSCs), and M2-polarized tumor-associated macrophages (TAMs) [34,35,36,37]. These cells secrete immunosuppressive cytokines like TGF-β and IL-10, express inhibitory ligands, and interfere with T cell homing, all of which collectively impair CAR T cell function in the TME and are rarely accounted for in standard in vitro assays [38,39]. Conversely, by releasing pro-inflammatory cytokines and chemokines, CAR T cells can also recruit other immune and stromal populations to the TME [40]. This is particularly true for fourth generation CAR T cells, which are engineered to secrete immunomodulatory factors that enhance not only their own anti-tumor capabilities but also that of neighboring immune cells [41].

**Table 1:** Summary of characteristics for in vitro modeling of CAR T cells.

Characteristics	In Vitro Modeling Summary
Experimental design/setup	CAR T cells co-cultured with antigen-expressing tumor cell lines or plate-coated antigen
Physiological/ immune complexity	Highly reductionist with limited biological complexity due to simple co-culture of CAR T cells and target cell lines or antigen
Tumor microenvironment (TME)	Largely absent; minimal immunosuppressive signaling [34,35,36,37]
CAR T activation	Activation marker (e.g., CD25, CD69, CD71, HLA-DR) expression measured by flow cytometry after co-culture [16]
CAR T exhaustion	Inhibitory receptor (e.g., PD-1, LAG-3, TIM-3, TIGIT, etc.) expression measured by flow cytometry after co-culture [17,18,19,20]
CAR T cell memory	T_N_, T_SCM_, T_CM_, T_EM_, T_EFF_ subsets can be determined by flow cytometry (markers: CCR7, CD45RA, CD45RO, CD62L) after co-culture [21]
Secretory profiling	Supernatant analysis for cytokines, chemokines, and cytotoxic granules by ELISA, CBA, Luminex, MSD [24,25]
Proliferative capacity	CFSE/CellTrace dilution, [3H]-thymidine incorporation, Ki-67 staining, BrdU incorporation, or cell counting [30]
Cytotoxicity	Direct killing assays (e.g., flow cytometry, luciferase/GFP tracking) or indirect assays (e.g., LDH, ATP release, impedance assays) [31]
Trafficking and infiltration	Not modeled
Evaluation of toxicity	Limited ability; largely restricted to quantitative measurements [32]
Experimental duration	Short-term (hours to days)
Cost and accessibility	High throughput, rapid, and relatively inexpensive
Primary advantage	Rapid evaluation of CAR T cell function and efficacy
Limitations	Poor representation of physiological tumor conditions (i.e., immunosuppressive TME, hypoxia, low pH), immune cell trafficking, and interactions with non-tumor cells (including other immune cell populations) [32,33,34,35,36,37,38,39,40,41,42,43,44,45]

Furthermore, in vitro assays typically fail to replicate other critical features of the TME, such as hypoxia, acidic pH, nutrient depletion, and abnormal vasculature (e.g., fibrotic stromal barriers), all factors that impact CAR T cell trafficking, persistence, metabolism, and overall effector function [42,43].

As a result, conventional 2D in vitro assays measuring cytotoxicity, proliferative capacity, and cytokine release, while important for proof-of-concept validation, demonstrate limited predictive value for in vivo therapeutic efficacy and systemic toxicities [44]. These reductionist models fail to capture key challenges observed in in vivo settings, particularly in solid tumors, and frequently overestimate anti-tumor effects, consequently resulting in suboptimal correlation with clinical outcomes [45].

To improve the translational relevance of 2D in vitro assays, future studies should consider incorporating stromal, endothelial, and immunosuppressive immune cells, introducing physical barriers to mimic tumor vasculature, and simulating hostile conditions (e.g., hypoxia, low pH, and nutrient scarcity). While in vitro models are important for early-stage CAR T cell evaluation, their inability to recapitulate the complexities of human physiology and the TME limits their translational value and underscores the need for complementary in vivo modeling.

## 3. In Vivo Characterization of CAR T Cell Efficacy

**Figure 2:**
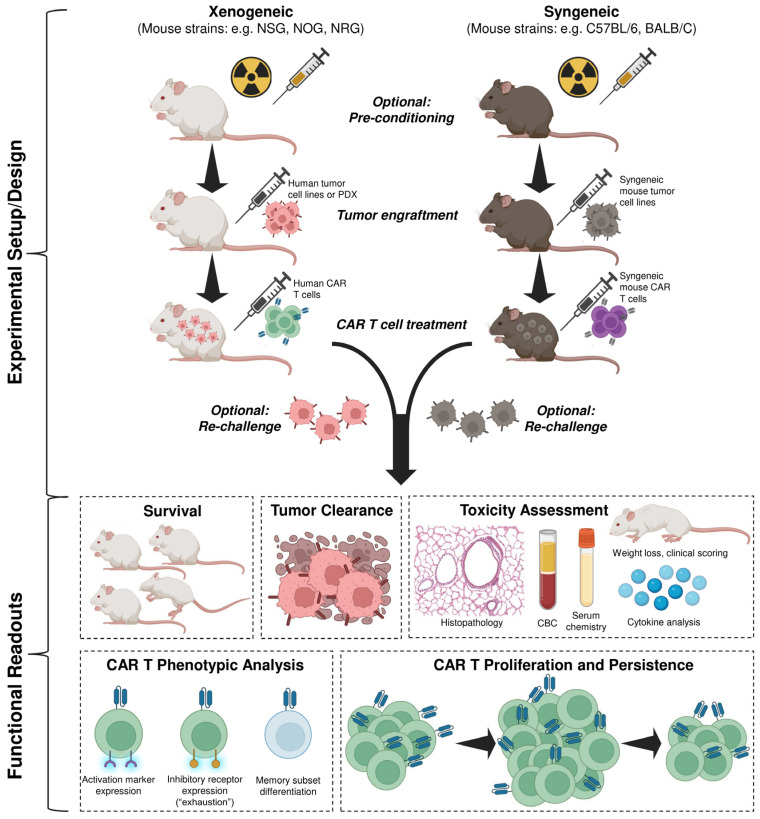
In vivo modeling of CAR T cell effector function, persistence, and anti-tumor efficacy typically begins with xenogeneic or syngeneic mouse models. Mice may be administered low-dose total body irradiation (TBI) or chemotherapy before tumor implantation to mimic clinical lymphodepletion. For xenogeneic studies, mice are implanted with human tumor or PDX cell lines and treated with human CAR T cells, while for syngeneic studies mice receive mouse tumor cell lines and are treated with mouse CAR T cells. Mice may be re-challenged to assess durability of CAR T cell responses. Anti-tumor efficacy is monitored via survival analysis and tumor burden/clearance, and systemic toxicities by body weight, clinical scoring, complete blood counts (CBCs), and serum chemistry (e.g., ALT, AST). CAR T cells are characterized post-infusion for phenotype (activation, exhaustion, memory) and long-term persistence.

In vivo animal models provide an essential platform for evaluating CAR T cell efficacy, biodistribution, such as persistence and trafficking, and safety in a physiologically relevant context. These models typically involve engrafting either human (xenogeneic) or mouse (syngeneic) tumor cell lines into mice and subsequently treating them with either human or mouse CAR T cells respectively to evaluate anti-tumor efficacy, CAR T cell phenotype (activation, exhaustion, memory) and expansion, and systemic toxicities (Figure 2). Along with mouse models, large animal models, such as canines and non-human primates (NHPs), are sometimes used due to their closer immunological and also physiological resemblance to humans, ultimately the targeted treatment group.

Unlike in vitro assays, in vivo models require CAR T cells to circulate systemically and traffic to tumor sites where they interact with the various stromal and immune components of the tumor microenvironment (TME) and vasculature. These interactions are often quite complex and can greatly affect therapeutic efficacy, CAR T cell persistence and durability, and toxicity and also inform mechanisms of resistance, all of which cannot be recapitulated in reductionist in vitro cell culture systems.

### 3.1. Xenogeneic Mouse Modeling

The overwhelming number (>90%) of CAR T cell preclinical studies use xenogeneic mouse models [46]. Xenogeneic models typically involve implanting human tumor cell lines or patient-derived xenografts (PDXs) into severely immunodeficient mice, such as NSG, NOG, or NRG strains, and subsequently treating them with human CAR T cells [47,48]. These mice lack functional T, B, and NK cells and exhibit deficiencies in their macrophage and dendritic cell compartments, allowing for engraftment of both human tumor cells and human CAR T cells [49].

#### 3.1.1. Tumor Cell Lines Versus Patient-Derived Xenografts

While cell lines are easy to culture and are relatively homogenous, they typically express uniformly high levels of the target antigen, which does not accurately reflect antigen heterogeneity typically seen in patient tumors [50]. PDXs, generated from clinical tumor samples grown and passaged in immunodeficient mice, may improve translational relevance by modeling tumor heterogeneity although they are limited logistically by availability (depending on the tumor), long establishment times, and high costs. Repeated passaging also selects for dominant fast-growing subclones thereby reducing tumor heterogeneity over time [51]. In addition, the human TME in PDXs is progressively replaced with murine extracellular matrix and stromal cells, limiting its ability to faithfully model the human TME [52]. This stromal replacement alters the PDX vasculature and also affects chemokine gradients, which can significantly affect CAR T cell adhesion and extravasation, and infiltration [53].

#### 3.1.2. Experimental Workflow

Hematological tumors are typically injected intravenously while solid tumors can be implanted subcutaneously, orthotopically, or intravenously, depending on the cancer type and the experimental objectives. After a period of engraftment, CAR T cells are then administered intravenously, and anti-tumor efficacy is monitored primarily through survival analysis over time. To better mirror the clinical process, lymphodepleting regimes, such as radiation and chemotherapy (e.g., cyclophosphamide/fludarabine), may be administered beforehand to enhance CAR T cell engraftment but this is not usually applied given the mice are lymphopenic. This preconditioning, however, can lead to altered tumor metabolism and premature tumor clearance, complicating interpretation of CAR T cell efficacy as well as affecting potential toxicities [54]. Furthermore, since immunodeficient mice lack other functional immune populations and interactions between them and CAR T cells, the effects of lymphodepletion in this context may not be physiologically representative [55].

#### 3.1.3. Assessment and Key Readouts

Tumor burden can be assessed via caliper measurements (solid tumors), flow cytometry, MRI, or bioluminescent/fluorescent imaging using tumor cells engineered to express luciferase or fluorescent proteins [56,57]. These imaging techniques are especially valuable for tracking disease progression in disseminated or metastatic cancer models.

Additionally, CAR T cell persistence and memory phenotype (T_N_, T_SCM_, T_CM_, T_EM_, T_EFF_) can be evaluated by collecting and processing blood, lymphoid tissues (although lymph nodes are absent in most immunodeficient strains), tumor-infiltrated organs, and tumors themselves and analyzing via flow cytometry or immunohistochemistry. Flow cytometry analysis can also be used to determine the extent of exhaustion by analyzing inhibitory receptor expression. Furthermore, newer approaches such as TCR sequencing and single-cell transcriptomics can further allow detailed characterization of CAR T cell clonal dynamics along with phenotype analysis and memory differentiation in vivo, providing novel insights into specific subsets that contribute to a durable response.

Cytokine and chemokine secretion profiles can also be evaluated by serum analysis through ELISA, CBA, or multiplex platforms such as Luminex and MSD, for both human and mouse cytokines. For toxicity assessments, weight monitoring (for significant weight loss) and clinical scoring are commonly used as initial indicators for overall health post-CAR T cell infusion [58]. Complete blood counts can be performed to measure for hematological changes [59]. In parallel, serum chemistry testing can be used to monitor and evaluate the health and function of specific organs, such as measuring levels of alanine aminotransferase (ALT) and aspartate aminotransferase (AST) for liver injury, alkaline phosphatase and bilirubin for biliary dysfunction, creatine and blood urea nitrogen (BUN) for kidney damage, and electrolyte panels (e.g., sodium, potassium, chloride, calcium, magnesium) for metabolism and fluid balance [59,60]. Additionally, organs can be formalin-fixed, paraffin-embedded, sectioned, and stained (e.g., hematoxylin and eosin (H&E)) to determine CAR T tissue infiltration and potential immunopathology.

#### 3.1.4. Limitations of Xenogeneic Mouse Modeling

While immunodeficient mouse models provide valuable insight into CAR T cell efficacy and persistence in vivo, they ultimately lack a functional immune system and therefore cannot accurately model the full spectrum of CAR T cell interactions with other immune cell types. Furthermore, while functional MDSCs and TAMs have been detected in NSG mice, their impact on human CAR T cells may be minimized due to species incompatibility [61].

To better model these human immune interactions, humanized mouse models can be generated by engrafting human PBMCs or hematopoietic stem cells into immunodeficient mice. To promote engraftment and support development of human immune lineages, these mice are often engineered to express additional human components such as cytokines (e.g., NSG-SGM3), MHC molecules, or phagocytosis-inhibiting receptors like SIRPα (e.g., MISTRG mice). Despite these advances, however, cross-species incompatibilities remain. Human CAR T cells cannot fully interact with mouse cytokines, chemokines, vasculature, or stroma, and additionally, humanized mice develop graft-versus-host disease (GvHD), limiting experimental windows and precluding long-term study of tumor relapse and the efficacy of repeated dosing [62]. This is especially critical as 30 to 60% of patients with B-ALL treated with CD19-targeting CAR T cells experience relapse, including 10–20% with CD19-negative disease, while relapse rates commonly exceed 80% in multiple myeloma (MM) patients treated with BCMA-targeting CAR T cells [63,64]. In addition, long-term modeling is necessary to assess the potential of serious safety events, such as secondary malignancies associated with CAR transgene integration, which, while rare, have been reported in treated patients [65]. The inability of xenogeneic mouse models to model relapse, potential tumor resistance and antigen escape, multiple CAR T cell infusions, and potential secondary malignancies creates a translational gap in bridging preclinical studies to clinical outcomes.

Furthermore, while human cytokines such as TNF and IL-1α/β can signal through mouse receptors, cytokines such as GM-CSF and IFN-gamma are species-specific. Thus, xenogeneic models are inconsistent and unreliable for evaluating systemic toxicities with mixed reports showing systemic toxicities in some cases and not in others [66,67,68,69]. In human patients treated with CAR T cells, CRS is driven primarily by cytokines produced by monocytes and macrophages, particularly IL-6, with both immune cells being activated downstream of CAR T cell activation (predominantly through GM-CSF and TNF production) [70,71]. Many xenogeneic models, however, lack a fully functional myeloid compartment and, in addition to poor cross-reactivity when human CAR T cells are activated and secrete human cytokines, elevated releases of monocyte- and macrophage-derived cytokines are mostly absent [72]. As a result, immunodeficient mice fail to recapitulate systemic toxicities, such as CRS and ICANS.

This limitation is particularly important in the case of ICANS, which was initially unexpected in patients receiving CD19-targeting CAR T cells given that CD19 expression is largely exclusive to B-lineage cells and mostly absent from cells of the central nervous system. Rather than resulting from OTOT toxicity, ICANS, which typically follows CRS, is driven by systemic inflammation that then results in endothelial activation, vascular permeability, and blood–brain barrier disruption [73]. This sequence of events enables a large amount of cytokines and even CAR T cells to enter the cerebrospinal fluid (CSF) where they then activate microglia cells and propagate a neuroinflammatory cascade that results in patient symptoms ranging from mild, such as aphasia and impaired motor skills, to severe, such as seizures and cerebral edema [73,74]. The inability of xenogeneic mouse models to accurately reproduce these inflammatory cascades observed clinically not only limits their predictive value for CRS and ICANS but also raises the possibility that other additional toxicities may go unnoticed during preclinical evaluation.

Humanized mouse models may address this key limitation in part by the introduction of human myeloid cells, but still, differences in engraftment, cytokine production, and cross-species signaling (human cytokines in a mouse) limit accurate modeling of these toxicities. Furthermore, differences in endothelial adhesion molecule expression and chemokine signaling between species may confound evaluation of CAR T cell trafficking in xenogeneic models [75].

Another critical challenge limiting xenograft models in the evaluation of human CAR T cells is that currently approved CAR T cell therapies are autologous, whereas preclinical studies often involve CAR T cell interactions with allogeneic tumor lines in xenogeneic recipients prior to clinical application, which may yield distorted or inaccurate outcomes relative to the clinical scenario.

### 3.2. Syngeneic Mouse Modeling

In syngeneic models, mouse tumor cell lines are implanted into immunocompetent mice, typically C57BL/6 or BALB/c strains, which are then treated with mouse-derived CAR T cells. Only roughly 4% of preclinical CAR T studies use syngeneic models [46]. However, these models offer several distinct advantages over xenogeneic ones particularly by enabling the evaluation of CAR T cell function in the context of a fully intact immune system (Table 2). This includes interactions and crosstalk with endogenous T cells, innate immune cells, stromal components, and tumor vasculature, all of which can modulate CAR T cell efficacy, expansion, and persistence [76,77].

#### 3.2.1. Key Advantages

In particular, this allows syngeneic models to better model systemic toxicities such as CRS and ICANS, as cytokines are species-compatible and the myeloid compartment remain intact [72]. Furthermore, OTOT effects arising from CAR T cell treatment can be more accurately evaluated, as targeted TAAs are also naturally expressed on healthy tissues without the issue of antigen cross-species reactivity [79]. Similar to xenogeneic mouse modeling, body weight monitoring, clinical scoring, CBC, serum chemistry testing, and histopathological analysis can be used for the evaluation of toxicity [58,59,60].

Additionally, while lymphodepleting regimens can be applied in xenogeneic models, their use in syngeneic ones provides greater physiological relevance owing to an intact immune system [55]. Lymphodepletion reduces the population of immunosuppressive immune cells, such as regulatory T cells and myeloid-derived suppressor cells, and also removes “cytokine sinks”, endogenous NK and T cells, allowing for CAR T cells to fully utilize effector cytokines such as IL-2, IL-7, IL-15, and IL-18 [80]. Furthermore, lymphodepletion can upregulate expression of certain TAAs, such as B7-H3, and integrins, such as intracellular adhesion molecule-1 (ICAM-1), leading to increased CAR T cell infiltration into the tumor. Tumor cell death can also be induced by causing an increase in antigen release, which is subsequently captured by antigen-presenting cells (APCs), which can then enhance CAR T cell activation [81]. Plus, lymphodepletion may also increase co-stimulatory molecule expression, such as CD80, on APCs [82].

Furthermore, syngeneic models may represent a better model for studying CAR T cell trafficking, which is extremely important in determining therapeutic efficacy especially against solid tumors. Clinically, treatment against a solid tumor requires chemokine-directed homing of the CAR T cells to adhere to activated endothelium, undergo transendothelial migration, and localize within the TME. Mechanistically, trafficking efficiency requires strong chemokine receptor expression (e.g., CXCR3, CCR5, CCR2) and integrin activation. A study demonstrated that CXCR3 expression along with its ligands CXCL9 and CXCL10 are necessary for T cell trafficking into tumor tissues in mice [83].

Additionally, syngeneic models enable detailed characterization of CAR T cell phenotype, persistence, and memory differentiation within a fully functional immune system. In particular, as immune populations recover post-lymphodepletion, these models allow assessment of how CAR T cells compete with endogenous cells for cytokines. This is especially valuable for modeling repeated dosing or long-term responses, which are limited in xenogeneic models due to GvHD or cross-species immune incompatibility.

#### 3.2.2. Limitations of Syngeneic Mouse Modeling

However, translating syngeneic models to humans is limited by reliance on mouse CAR T cells and tumor antigens, which may not accurately reflect the functionality of human CAR T cells nor their interactions with corresponding tumor antigens. Additionally, mouse tumor cell lines are often more immunogenic than patient tumors, which may cause overestimation of CAR T cell potency [84]. Therefore, while syngeneic models are important to study immune dynamics and safety profiles, their results should be evaluated together with xenogeneic and humanized models for a more comprehensive preclinical evaluation.

### 3.3. Overall Limitations of In Vivo Mouse Models

While in vivo models have significantly advanced the preclinical evaluation of CAR T cells, mouse biology does not fully capture human physiology particularly in terms of immune cell trafficking, cytokine signaling, and systemic toxicity. In xenogeneic models, this limitation is particularly pronounced since human CAR T cells must interact with endogenous mouse endothelial adhesion molecules, chemokines, and cytokines, and vice versa, where cross-species reactivity is often only partial or absent. However, even in syngeneic mouse models, important differences between mice and humans can complicate clinical translation from preclinical results. For example, while many human chemokines and their corresponding receptors have homologues in mice with similar functions, their expression patterns and tissue distribution can differ substantially between species and, moreover, certain chemokines are expressed in humans but not mice (e.g., CCL13, IL-8/CXCL8, CCL14, CCL18) while others are present in mice but not in humans (e.g., CCL12, CXCL15) [78]. These differences may alter how immune cells, including CAR T cells, are recruited and localized, along with their inflammatory responses, making them not fully representative of human disease. Additionally, architectural differences in immune organs, such as the spleen and gut-associated lymphoid tissue, can influence lymphocyte circulation and tissue persistence, potentially affecting CAR T cell biodistribution and functionality [85,86].

Furthermore, most preclinical mouse modeling of CAR T cell efficacy relies on young, lean mice usually of a single sex, which fails to account for patient-intrinsic variables, such as sex, age, or metabolic state (e.g., obesity), further limiting their translational relevance. Emerging research shows that these factors are not trivial and can potentially influence the phenotype of the final CAR T cell product and affect efficacy, toxicity profiles, and durability of responses. In a large-scale multivariate analysis of 2928 patients treated with CAR T therapy, while there were no differences in hospital outcomes, such as early mortality, 30-day readmissions, and discharge disposition, between male and female patients, female patients had twice the odds of developing leukopenia but 40% lower odds of developing acute kidney injury [87]. In a separate cohort of 432 patients who received CD19-targeting CAR T cell therapy for LBCL, male patients demonstrated a significantly greater risk of relapse correlated into lower progression-free survival (PFS) [88]. These findings suggest that sex may affect toxicity profiles and long-term efficacy although it is rarely modeled as a factor preclinically.

Age is another underdiscussed variable affecting CAR T cell responses. Retrospective studies have yielded mixed results with some reporting that CAR T cell therapies are equally efficacious with comparable PFS, hospital outcomes, and toxicity across all age groups while other studies suggesting superior clinical outcomes in pediatric and young adult patients [89,90,91,92]. However, aging is characterized by chronic low-grade inflammation (“inflammaging”), cellular senescence, and mitochondrial dysfunction, all of which can impair antigen-specific T cell responses [89,93]. In accordance, a study demonstrated that mouse HER2- and CAR19-targeting CAR T cells from aged mice demonstrated a significant decline in nicotinamide adenine dinucleotide (NAD), a key metabolite for mitochondrial function, increased DNA damage, and the inability to maintain T_CM_ cells, resulting in a large decrease in in vivo anti-tumor efficacy [94]. These findings align with previous literature showing that aging leads T cells to shift toward terminal differentiation, increased exhaustion, reduced persistence, and decreased anti-tumor responses [92,95]. Additionally, aging reshapes the TME, leading to increased numbers of immunosuppressive cells, such as MDSCs, Treg cells, and M2-like macrophages, which secrete TGF-β, IL-10, and reactive oxygen and nitrogen species that can further promote CAR T cell exhaustion and dysfunction [96]. Nevertheless, age as a variable influencing CAR T cell efficacy is often unaccounted for in preclinical modeling.

Similarly to aging, obesity similarly is marked by metabolic dysregulation and chronic inflammation that can lead to reduced T cell effector function and greater exhaustion [97]. Furthermore, leptin signaling from excess adiposity may further upregulate PD-1, exacerbating T cell exhaustion and dysfunction [98]. Although obesity initially promotes recruitment of pro-inflammatory Th1, Th17, and CD8^+^ T cells into adipose tissues, this chronic stimulation ultimately drives premature exhaustion and greatly diminished pro-inflammatory cytokine production resulting in impaired anti-tumor responses [99,100]. Interestingly, despite enhanced tumor growth and T cell dysfunction, obese patients are reported to have improved responses to immune checkpoint inhibitors, a phenomenon termed the “obesity paradox” [101]. In the context of CAR T cell therapy, the correlations between body mass index (BMI) and clinical outcomes and toxicity remain incomplete. Several studies have reported conflicting results, with one demonstrating no association between BMI and CAR T cell efficacy or toxicity, another suggesting worse outcomes in overweight patients compared to normal and obese patients, and yet another reporting improved three-year survival and lower rates of infection in patients with high BMI [102,103,104]. Another study reported higher BMI was associated with earlier onset of CRS and increased probability of developing grade ≥ 2 CRS [105]. Despite these conflicting clinical results, a recent preclinical study demonstrated in a preclinical model of triple negative breast cancer that, while mouse B7-H3-targeting CAR T cells from control and diet-induced obesity (DIO) mice showed similar in vitro tumor efficacy, CAR T cells from DIO mice had impaired glycolytic capacity, reduced tissue-resident memory T cell (TRM) formation, and diminished in vivo efficacy [106]. Perhaps more notably, it further shows that CAR T cells generated from age- and diet-matched mice show greater anti-tumor efficacy in control tumor-bearing mice compared to DIO counterparts [106]. These results indicate that obesity not only has direct effects on T cell metabolism but also broadly and systemically alters the host immune and inflammatory landscapes, ultimately impairing anti-tumor responses. Yet despite these findings, the effects of obesity in CAR T cell therapy are rarely accounted for and modeled preclinically, overlooking a clinically important metabolic state that may influence efficacy and toxicity.

Finally, toxicities observed in mice also do not always translate to humans, especially since disease states in mice must often be artificially induced. Neither tumor cell lines nor PDXs can fully capture the heterogeneity, TME, and complexity of human patient tumors, limiting the ability of these models to adequately simulate mechanisms of tumor resistance such as antigen loss or immune escape [107].

Altogether, these limitations highlight the fact that, while mouse models are effective in generating preliminary data regarding efficacy and possibly safety, they fail to faithfully reproduce the full complexities of human biology that can influence CAR T cell responses and toxicity profiles in patients. As a result, the predictive ability of in vivo mouse modeling towards clinical response remains inconclusive at best. In a meta-analysis of 303 preclinical mouse studies incorporating over 20 variables including tumor type, antigen target, cell line, mouse strain, preconditioning regimen, CAR design, and reported efficacy and toxicity, and correlating it to data from 105 corresponding clinical trials, machine learning models were unable to reliably predict clinical outcomes based on preclinical data alone, and high predictive accuracy was achieved only when tumor type was included as a feature [46]. In particular, strong preclinical results have demonstrated translational efficacy predominantly in hematological tumors, whereas multiple CAR T cell therapies targeting solid tumors across diverse antigens, including B7-H3, GD2, HER2, mesothelin, and PSMA, have shown robust preclinical efficacy yet failed to achieve objective responses in clinical trials [46,108]. Furthermore, while preclinical studies often report comparable efficacy across different target TAAs, in reality, clinical responses vary drastically depending on the specific TAA and the disease context [46]. Additionally, toxicity is highly underrepresented in preclinical models and results. In the same meta-analysis, only 4% of studies reported systemic toxicities whereas clinical incidences of CRS and ICANS are considerably higher and are reported at over 60% and 30% respectively depending on the study [109,110]. Overall, these findings demonstrate the limitations of using current mouse models to accurately predict clinical efficacy and safety.

### 3.4. Large Animal In Vivo Models

Large animals are far more anatomically, physiologically, and immunologically similar to humans than mice are and therefore a much more clinically translatable model to study cancer and immunotherapies. Their larger body size permits clinically relevant data regarding dosing strategies and infusion routes. Additionally, large animals have longer life spans allowing for them to be monitored longitudinally over many years. To address the inherent limitations of mouse modeling, researchers have increasingly used canine and NHP systems to study CAR T cell biology in vivo.

Canines are another large animal model with high translational value owing to their genetic and histological similarity to humans, comparable disease inheritance patterns and microbiome compositions, and shared environmental exposures [111]. Furthermore, cancers in dogs arise spontaneously in immunocompetent hosts, generating tumor heterogeneity along with tumor–immune interactions that more closely reflect human cancers than transplanted mouse tumors [112]. Additionally, canines and humans share similar enriched genes and oncogenic pathways that are involved in tumor etiology [113,114]. These comparable attributes have led researchers to explore the development of canine-specific CAR T cells both for therapeutic and translational value. In a study evaluating canine CD20-targeting CAR T cells to treat dogs with spontaneous DLBCL, researchers observed the relapse of CD20-negative antigen escape along with the development of anti-CAR antibodies, both of which were associated with the loss of CAR T cell persistence [115]. These are key findings that mirror clinical observations in human CAR T cell therapy but are difficult to model in mice. Furthermore, CAR T cell expansion in dogs treated with CAR T cell therapy significantly correlated with overall survival, validating clinical data in humans demonstrating that proliferative expansion is a key criterion determining therapeutic success [8,115]. Additionally, comparative oncology studies have shown that osteosarcoma (OS) is similar between canines and humans with closely aligned mutational burdens and tumor microenvironmental immune signatures [116,117]. Along those lines, canine B7-H3-targeting CAR T cells are being evaluated in treating dogs with spontaneous osteosarcoma where findings indicate that cytokine profiling, biomarkers (e.g., MCP-1 levels), and mild hepatotoxicity all parallel those observed in human clinical trials [118]. These studies highlight the translational value of canine models in capturing immune dynamics, antigen escape, and toxicity profiles comparable to human clinical CAR T cell therapy.

NHPs, typically rhesus macaques, are even more genetically, immunologically, and physiologically similar to humans. They share key developmental pathways and have been shown to recapitulate CAR T cell expansion kinetics and phenotype along with key CAR-T-cell-associated toxicities such as CRS and neurotoxicity with high fidelity observed in human CAR T cell clinical trials [119]. In a macaque CD20-targeting CAR T cell model, researchers reproduced the key features of human CAR T therapy, namely, B cell aplasia, CAR T cell expansion, and cytokine profiles [120]. Furthermore, they also were able to identify and characterize an activated population of bystander CD8+ cytotoxic T cells that had transcriptional signatures overlapping those seen in human patients, demonstrating the ability of NHP models to capture these complex immune network interactions that are otherwise elusive in mouse systems [120]. NHP models are also important for evaluating OTOT toxicities due to shared tissue expression patterns of certain antigens between macaques and humans. For example, receptor tyrosine kinase-like orphan receptor 1 (ROR1) shows comparable expression—high in B cell precursors and low expression in certain normal tissues like adipose, pancreas, and lung—across both macaques and humans [121]. However, evaluation of ROR1-targeting CAR T cells in NHPs demonstrated depletion of endogenous ROR1+ B cells without strong clinical toxicities, even at high doses, against normal tissue, therefore supporting positive safety assessment in a clinically translatable model [121]. Despite the advantages of NHP models, unlike canines, spontaneous tumor development is rare in NHPs, and there is a lack of well-characterized syngeneic tumors [72]. Furthermore, tumor induction is expensive, and logistically and ethically very challenging, which restricts the extent of preclinical modeling for cancer [122,123].

Ultimately, while canine and NHP models provide superior physiological and immunological relevance compared to mouse models and thereby enhance the predictive value of clinical outcome and toxicity assessments, their logistical and ethical constraints limit their scalability and widespread application. As such, large animal studies are at best translational platforms that are complementary to preclinical studies in mice and bridge the gap to early-phase human clinical trials, rather than as actual replacements for established mouse models. Furthermore, regulatory policies are seeking to minimize the reliance on animal experimentation altogether. Public and legislative pressure has catalyzed the development of new advanced human preclinical platforms designed to faithfully model tumor–immune cell dynamics while improving scalability, reproducibility, and clinical translation.

## 4. Future Technologies

In 2013, European states adopted EU Directive 2010/63/EU strengthening ethical reviews of all animal studies. Furthermore, the European Parliament passed a resolution in 2021 calling for a plan to phase out all animal testing by 2035. In the United States, the FDA Modernization Act 2.0 was signed into law in 2022, removing the federal mandate for animal testing in drug approvals. More recently, the SPARE Act was introduced to Congress in March 2025, proposing to ban animal use in all federally funded research facilities, and the NIH announced in July 2025 that it would no longer fund research exclusively relying on animal models. With the research community quickly moving towards replacing in vivo models, it is necessary to develop advanced in vitro techniques that faithfully replicate complex in vivo biology.

Patient-derived tumor organoids (PDTOs) are derived from stem or progenitor cells of a patient’s tumor, embedded in an 3D extracellular matrix and differentiated into organ-like tissues that largely preserve the histological and molecular heterogeneity of the original tumor. While PDTOs do not yet fully recapitulate the tumor microenvironment (TME) or CAR T cell trafficking dynamics within the human body, they represent a 3D spatial model to study CAR T cell efficacy in a heterogeneous tumor. Multiple studies have demonstrated the strong predictive ability of PDTOs for immunotherapy outcomes [124,125]. Notably, a recent study successfully generated glioblastoma PDTOs that accurately mirrored patient responses to CAR T cell therapy, both treatment efficacy and potential neurotoxicity, in a simultaneous, side-by-side comparison [126].

Furthermore, to better simulate immune cell trafficking and the TME, organ-on-a-chip (OOC) and multi-organ-on-a-chip (MOC) platforms have been developed that use microfluidic technologies to create interconnected microchannels mimicking vasculature, enabling the flow of immune cells through tissue-like structures that incorporate organ-specific tissues along with stromal cells, endothelial cells, immune cells, and components of the extracellular matrix [127]. Along with their ability to evaluate CAR T cell efficacy in complex, physiologically relevant environments, these systems have been shown to successfully replicate interactions between CAR T and other cell types, such as M2-like TAMs impairing CAR T cell cytotoxicity [128]. Researchers have also engineered these platforms to emulate TME-associated stresses such as hypoxia and acidic pH, allowing for predictive assessment of CAR T cell function under adverse environmental conditions [129,130].

Complementing both established and innovative techniques are in silico modeling and machine learning (ML), which are transforming CAR T cell development. These computational tools can simulate molecular interactions and predict CAR-to-antigen binding interfaces at the atomic level, enabling the development of CAR constructs that avoid steric hindrance and improve target engagement [131,132]. ML algorithms can further integrate large datasets, including single-cell RNA sequencing (scRNA-seq), spatial transcriptomics, proteomics, and metabolomics, and link them to patient outcomes and uncover key cellular mechanisms, signaling networks, and predictive biomarkers influencing CAR T cell efficacy. In one study, deep learning models accurately predicted over 90% of clinical outcomes in mantle cell lymphoma patients [133]. In a recent study, researchers analyzed and integrated data from 256 patients covering a diverse range of clinical and biological data from multiple stages, including preinfusion flow cytometry phenotyping, baseline serum markers, CAR T cell ex vivo expansion and viability, postinfusion CAR T cell persistence and serum markers, and clinical response, and synthesized this all into a supervised machine learning framework to develop a model that identified pan-cancer predictive biomarkers and predicted favorable or unfavorable patient outcomes of CAR T cell therapy with a peak performance of 87.5% (AUROC) [134]. When computational approaches are integrated with experimental data, they can yield personalized, mechanistically predictive insights that improve translational success without reliance on animal models.

Overall, a comprehensive understanding of the range of preclinical models, including traditional in vitro and in vivo experimental designs as well as newer technologies, and their respective advantages and limitations, is essential for developing, validating, and translating CAR T cells from the bench to the clinic (Table 3).

**Figure 3:**
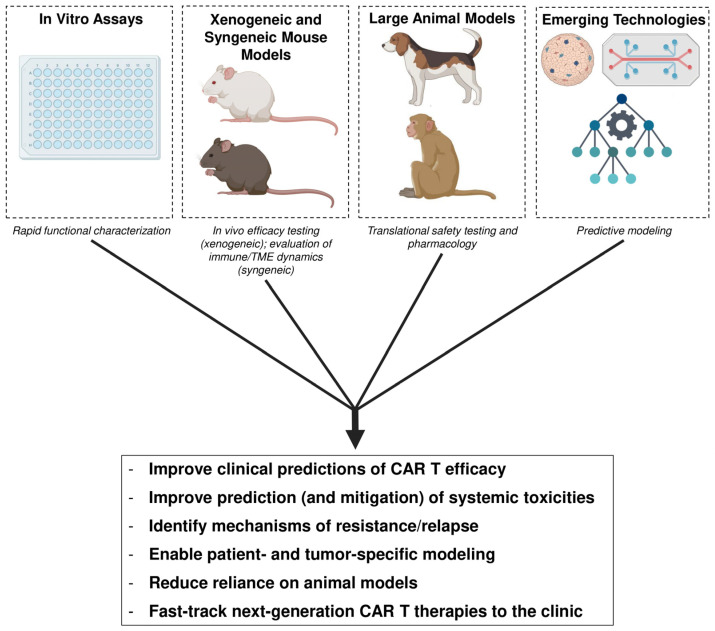
CAR T cell preclinical modeling encompasses in vitro studies, xenogeneic and syngeneic mouse modeling, large animal modeling, and emerging technologies all for the purpose of evaluating efficacy, safety, persistence, cellular interactions, and mechanisms of resistance. Integrating these workflows can enable better understanding of CAR T cell biology and greatly improve our ability to translate results from the lab to the clinic.

By generating robust data from these preclinical models and evaluating it holistically, researchers can gain deeper insights into the factors affecting CAR T cell efficacy and persistence, better predict clinical responses, including potential systemic toxicities, and identify mechanisms of resistance (Figure 3). Ultimately, this integrative and streamlined approach can help facilitate the development of new and improved CAR T cell products that address current challenges, accelerate their translation to the clinic, and one day possibly enable patient- and tumor-specific modeling and personalized CAR T cell therapies.

## 5. Conclusions

While in vitro assays offer a controlled platform for evaluating CAR T cell function, proliferation, and cytotoxicity, they cannot fully recapitulate the complexities of the TME or cross-immune cell interactions in a human patient. In vivo models, including xenogeneic/syngeneic, mouse, and large animal models, enable more physiologically relevant assessment, but their clinical predictive ability remains limited due to species differences, logistical constraints, and incomplete representation of human immune dynamics and patient-intrinsic characteristics. Newer technologies, such as patient-derived tumor organoids, organ-on-a-chip systems, and computational modeling, hold promise to bridge some of these gaps while reducing reliance on animal models. Integrating these traditional in vitro and in vivo workflows with innovative technologies can enhance translational predictability, inform the development of safer, more effective CAR T cell therapies in the near future, and ultimately enable patient- and tumor-specific modeling for personalized CAR T cell treatment.

## Figures and Tables

**Table 2:** Chart summarizing the key differences and applications of xenogeneic vs. syngeneic in vivo CAR T cell modeling.

Characteristics	Xenogeneic Mouse Models	Syngeneic Mouse Models
Experimental design/setup	Human tumor cell lines or PDX + human CAR T cells into immunodeficient (e.g., NSG, NOG, NRG) mice	Mouse tumor cell lines + mouse CAR T cells into immunocompetent (e.g., C57BL/6, BALB/c) mice
Endogenous immune system	Severely immunodeficient; lack functional T, B, and NK cells along with impaired myeloid populations [47,48,49]	Fully intact immune system with endogenous T cells, B cells, NK cells, macrophages, dendritic cells, etc.
Literature use	Very common (>90% of preclinical CAR T studies) [46]	Rare (~4% of studies) [46]
CAR product testing	Major advantage as enables testing of human CAR T cell constructs and products	Limited because CAR T cells and target tumor cells are derived from mice, not humans
Tumor microenvironment (TME)	Human TME even in PDX is progressively replaced by murine stroma and vasculature [52,53]	Full species-matched interactions between tumor, immune, and stromal cells recapitulating physiological interactions [76,77]
CAR T trafficking and infiltration	Limited due to cross-species chemokine and adhesion molecule incompatibilities [75,78]	Physiologically relevant due to species-matched chemokine, adhesion molecule, and integrin interactions [76,77]
CAR T immune cell interactions	Very limited interaction with endogenous immune cell populations and further limited by cross-species incompatibilities [61]	Full interaction with endogenous innate and adaptive immune cells [76,77]
Toxicity (CRS/ICANS) modeling	Poor due to lack of fully functional myeloid cells and cross-species cytokine signaling [72]	Better modeling of systemic toxicities due to fully functional myeloid cells and species-matched cytokine–receptor interactions [72]
On-target off-tumor toxicity	Difficult to evaluate due to cross-species differences in antigen protein sequence and tissue expression [79]	Can evaluate more accurately due to species-matched CAR–antigen interactions and natural expression in mouse tissues [79]
Lymphodepletion relevance	Less physiologically relevant as mice are already lymphopenic [55]	More physiologically relevant and resembles clinical conditioning; models endogenous immune cell populations recovering postconditioning [55,80]
Relapse modeling	Limited due to short experimental windows due to xenogeneic GvHD [62]	Better suited for studying long-term responses and repeated dosing
Cost and accessibility	Widely used and standardized although more expensive; PDX models expensive	Generally less expensive although requires establishing models
Overall translational relevance	Useful for testing human CAR T constructs and products but limited in toxicity modeling and recapitulating CAR T interactions with endogenous immune cell and the TME	Useful for studying CAR T interactions and dynamics with endogenous immune cells; better at modeling preconditoning and toxicity; limited in evaluating actual human CAR product

**Table 3:** Chart summarizing preclinical models for evaluating CAR T cells, their strengths and weaknesses, and what they are best suited for.

Model	Key Advantages	Key Limitations	Best Applications
2D In Vitro Assays	High throughput and inexpensive; fast functional characterization of CAR T phenotype and anti-tumor efficacy	Highly reductionist; lack CAR T cell interactions with tumor microenvironment (TME) and endogenous immune cell populations; trafficking not modeled	Early CAR T cell construct and product validation and characterization
Xenogeneic Mouse Models	Allows testing of human CAR T cell anti-tumor efficacy against human tumors and persistence in vivo; well-established and used in literature	Immunodeficient; cross-species incompatibilities limit human CAR T cell and cytokine interactions with endogenous mouse immune and stromal cells; poor modeling of CRS/ICANS	Initial in vivo anti-efficacy testing and characterization of CAR T cell persistence and biodistribution
Syngeneic Mouse Models	Intact immune system; species-compatible cytokine and chemokine signaling enabling evaluation of CAR T interactions with immune, stromal, and tumor microenvironment (TME) cells; allow studying trafficking, lymphodepletion effects, and systemic toxicities	Use mouse CAR T cells against mouse tumor antigens, making efficacy data less clinically relevant	Studying CAR T cell interactions with endogenous immune cell populations, stromal cells, and the tumor microenvironment; toxicity modeling
Large Animal Models (e.g., Canines, NHPs)	Much greater anatomical, physiological, and immunological similarity to humans; clinically relevant dosing; spontaneous tumors in canines; more accurate modeling of CAR T expansion and systemic toxicities in vivo	High cost, ethical constraints, limited tumor models, lack of scalability	Translational studies for validation of efficacy, dosing, and safety
Patient-Derived Tumor Organoids (PDTOs)	3D architecture; preserve patient tumor heterogeneity; strong predictability for patient responses	Cannot model CAR T trafficking	Personalized therapy testing, tumor resistance studies
Organ-On-A-Chip/Multi-Organ-On-A-Chip	Microfluidic system mimicking vasculature and immune trafficking; incorporates stromal and immune cells; no animal use	Technically complex and limited scalability	Studying CAR T trafficking and TME interactions without animal use
In Silico Modeling and Machine Learning	Integrate multiomics datasets; identify biomarkers and predict clinical outcomes; no animal use	Technically complex and dependent on published datasets	Biomarker discovery and patient response outcome prediction without animal use

## Data Availability

No new data was created and data availability is not applicable.

## Abbreviations

The following abbreviations are used in this manuscript:

ALT	Alanine aminotransferase
APC	Antigen-presenting cell
AST	Aspartate aminotransferase
ATP	Adenosine triphosphate
B-ALL	B cell acute lymphoblastic leukemia
BCMA	B-cell maturation antigen
BMI	Body mass index
BUN	Blood urea nitrogen
CBA	Cytokine bead array
CAR	Chimeric antigen receptor
CBC	Complete blood count
CCR	CC chemokine receptor
CD	Cluster of differentiation
CFSE	Carboxyfluorescein succinimidyl ester
CRS	Cytokine release syndrome
CXCR	CXC chemokine receptor
DIO	Diet-induced obesity
DLBCL	Diffuse large B cell lymphoma
E:T	Effector-to-target
ELISA	Enzyme-linked immunosorbent assay
FDA	Food and Drug Administration
GFP	Green fluorescent protein
GM-CSF	Granulocyte–macrophage colony-stimulating factor
GvHD	Graft-versus-host disease
H&E	Hematoxylin and eosin
HER2	Human epidermal growth factor receptor 2
ICAM-1	Intercellular adhesion molecule 1
ICANS	Immune-effector-cell-associated neurotoxicity syndrome
IFN-γ	Interferon gamma
IL	Interleukin
LDH	Lactate dehydrogenase
MCP-1	Monocyte chemoattractant protein-1
MDSC	Myeloid-derived suppressor cell
MHC	Major histocompatibility complex
MIP-1α	Macrophage inflammatory protein-1 alpha
ML	Machine learning
MM	Multiple myeloma
MOC	Multi-organ-on-a-chip
MRI	Magnetic resonance imaging
MSD	Meso Scale Discovery
NAD	Nicotinamide adenine dinucleotide
NHP	Non-human primate
NK	Natural killer (cells)
NOG	NOD/Shi-scid/IL-2Rγ^null^ (mouse strain)
NRG	NOD.Cg-Rag1^tm1Mom^ Il2rg^tm1Wjl^/SzJ (mouse strain)
NSG	NOD.Cg-Prkdc^scid^ Il2rg^tm1Wjl^/SzJ (mouse strain)
OOC	Organ-on-a-chip
OS	Overall survival
OTOT	On-target off-tumor
PBMC	Peripheral blood mononuclear cell
PDTO	Patient-derived tumor organoid
PDX	Patient-derived xenograft
PD-1	Programmed cell death protein 1
PFS	Progression-free survival
qPCR	Quantitative polymerase chain reaction
R/R	Relapsed or refractory
ROR1	Receptor tyrosine kinase-like orphan receptor 1
scRNA-seq	Single-cell RNA sequencing
SDF-1α	Stromal cell-derived factor 1 alpha
SIRPα	Signal regulatory protein alpha
TAA	Tumor-associated antigen
TAM	Tumor-associated macrophage
T_CM_	Central memory T cell
TCR	T cell receptor
T_EFF_	Effector T cell
T_EM_	Effector memory T cell
TME	Tumor microenvironment
T_N_	Naïve T cell
TNF	Tumor necrosis factor
T_reg_	Regulatory T cell
T_RM_	Residential memory T cell
T_SCM_	Stem cell memory T cell
TLS	Tumor lysis syndrome
